# 827 Application of Synthetic Electrospun Fiber Matrix as a Template for Wound Bed Preparation Before Autograft

**DOI:** 10.1093/jbcr/iraf019.358

**Published:** 2025-04-01

**Authors:** Nathan Cockerill, Philomene Spadafore, Suzanne Osborn, Karen Richey, Kevin Foster, Arpana Jain

**Affiliations:** Diane & Bruce Halle Arizona Burn Center; Diane & Bruce Halle Arizona Burn Center; Diane & Bruce Halle Arizona Burn Center; Diane & Bruce Halle Arizona Burn Center; Diane & Bruce Halle Arizona Burn Center; Diane & Bruce Halle Arizona Burn Center

## Abstract

**Introduction:**

Full-thickness tissue losses due to burn injury or soft tissue infection present a difficult challenge to reconstruction efforts. Application of split-thickness skin autograft remains the mainstay to heal a large area deep wound. However, the presence of a healthy wound bed is imperative for successful graft healing. We present a case series from a busy burn center where SEFM was used to successfully salvage complex wounds and ultimately provide a template for autograft application. SEFM is an alternative to xenogeneic and human allogenic skin substitute that is composed of resorbable electrospun fibers. It mimics the scale, structure, and architecture of native human tissue. The purpose of this review was to examine the efficacy of SEFM in treating complex wounds.

**Methods:**

We performed a retrospective review of all patients who received SEFM at a single burn center over a four-year period from 2020 to 2024. We tabulated the demographic information, burn injury characteristics, wound healing, surgical interventions, cost analysis and hospital outcomes.

**Results:**

The case series included 18 patients, 13 male and 5 female. The median age was 56.5, (range 18 to 71 years). The mean treatment area of SEFM application was 625 sq cm. The mean expense of the product was $7992. Of the 18 patients, 12 had successful autografting over the SEFM sites; one required reapplication of SEFM prior to grafting. Of the remaining patients who did not receive an autograft, 1 was transitioned to palliative care, 2 had amputations, 3 had wound healing without need for grafting. The average time to successful grafting was 17 days (range 2-36 days).

**Conclusions:**

We conclude that the SEFM application led to the development of a healthy wound bed in the majority of the patients treated at our burn center and ultimately supported the application of a successful autograft. The usual time to graft was 2-3 weeks after SEFM application.

**Applicability of Research to Practice:**

We present our experience with SEFM application in the reconstruction of full-thickness wounds and its efficacy in wound bed preparation before the application of autograft. Further studies are needed to define clinical parameters where SEFM may be deployed to create a dermal template in wound reconstruction.

**Funding for the Study:**

N/A

